# Supplementary material for: Hydrogen Bonding of Trialkyl-Substituted Urea in Organic Environment
Source: Molecules. 2025 Mar 21;30(7):1410. doi: 10.3390/molecules30071410 (PMC11990808; doi:10.3390/molecules30071410)
Supplement: Supplementary file 1 [file molecules-30-01410-s001.zip › molecules-3525051-supplementary (1).pdf]

Supporting information to

Hydrogen bonding in trialkyl substituted urea in organic environment

Zuzana Morávková, Jiří Podešva Valeria Shabikova, Sabina Abbrent, Miroslava Dušková-Smrčková

Institute of Macromolecular Chemistry, Czech Academy of Sciences, 162 00 Prague, Czech Republic

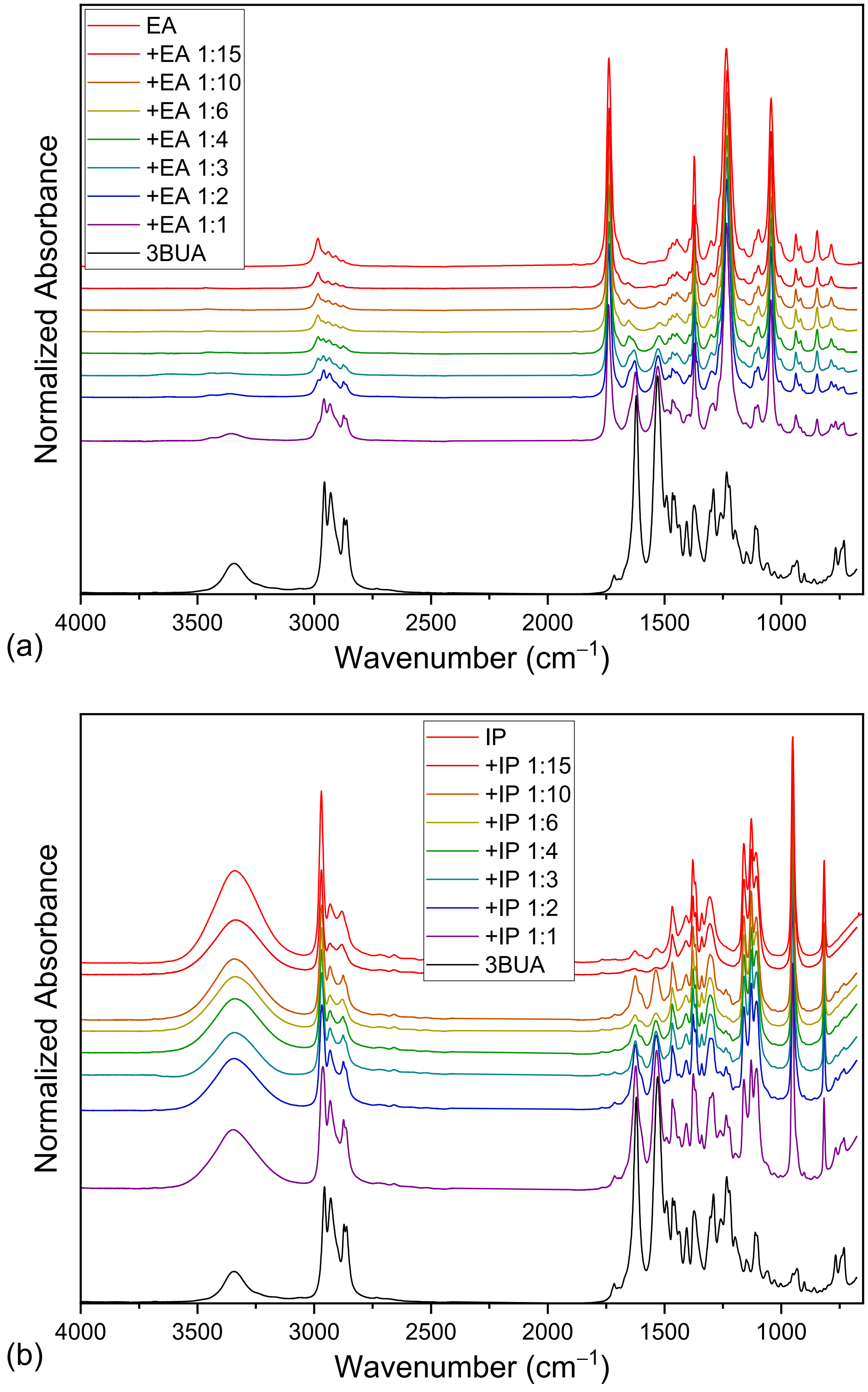

Figure S1. IR spectra of 3BUA under increasing dilution with (a) EA and (b) IP.

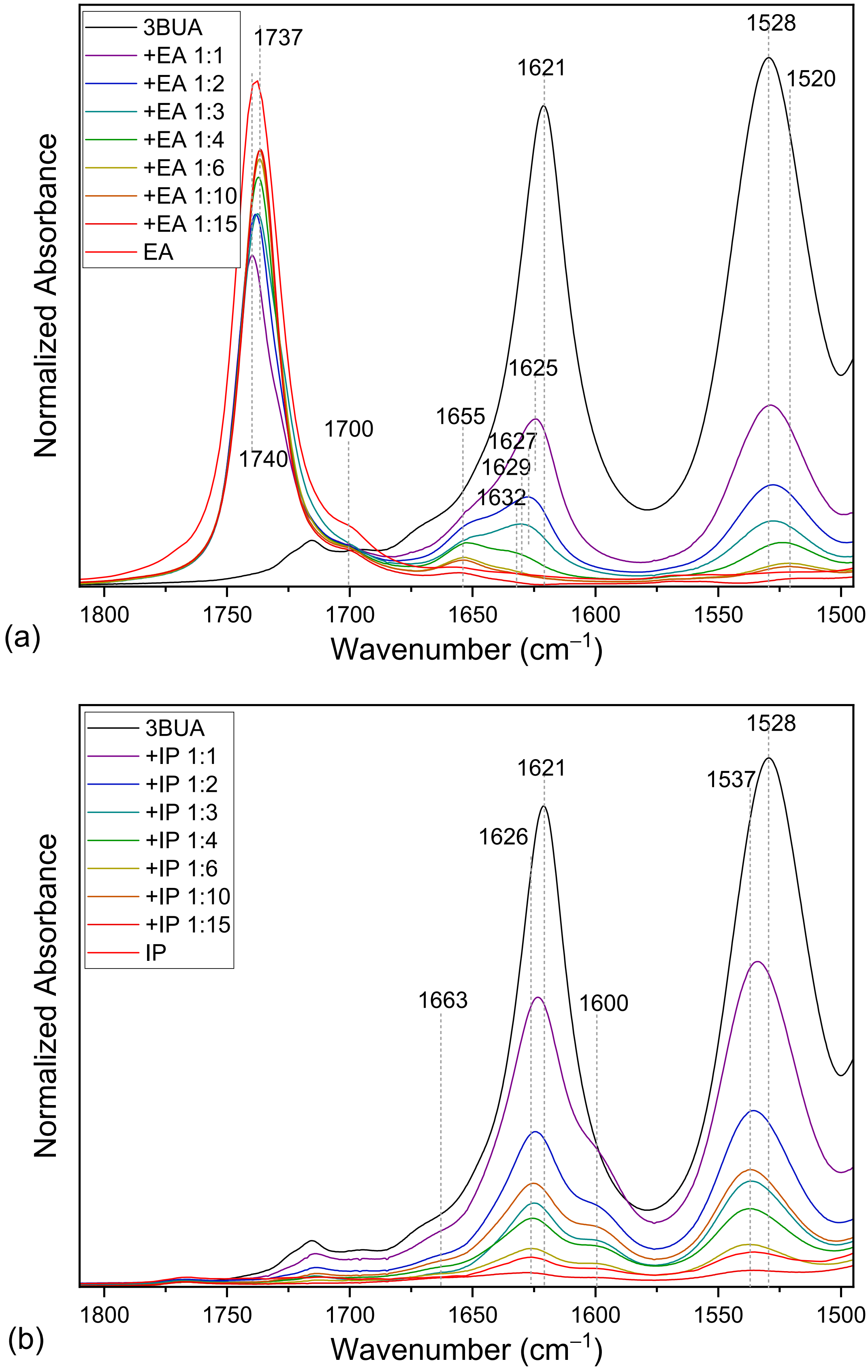

Figure S2. Carbonyl stretching and amide II region of the IR spectra of 3BUA under increasing dilution with (a) EA and (b) IP.

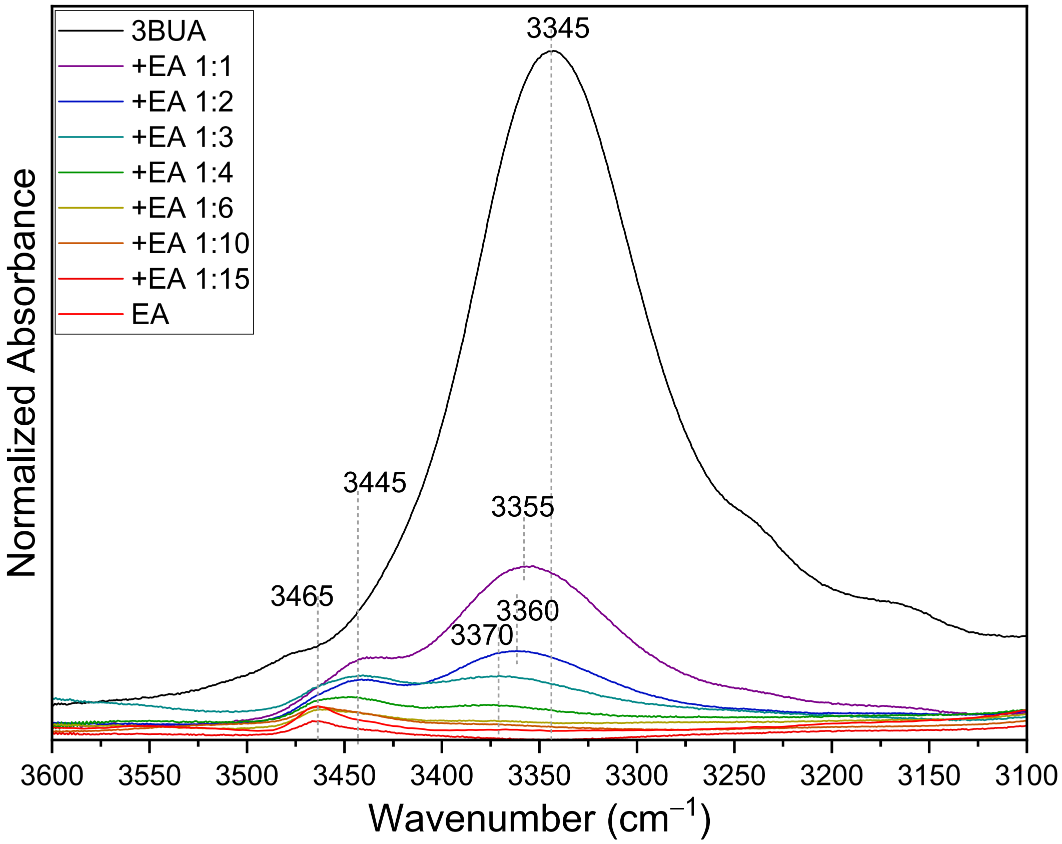

Figure S3. NH/OH stretching region of the IR spectra of 3BUA under increasing dilution with (a) EA and (b) IP.

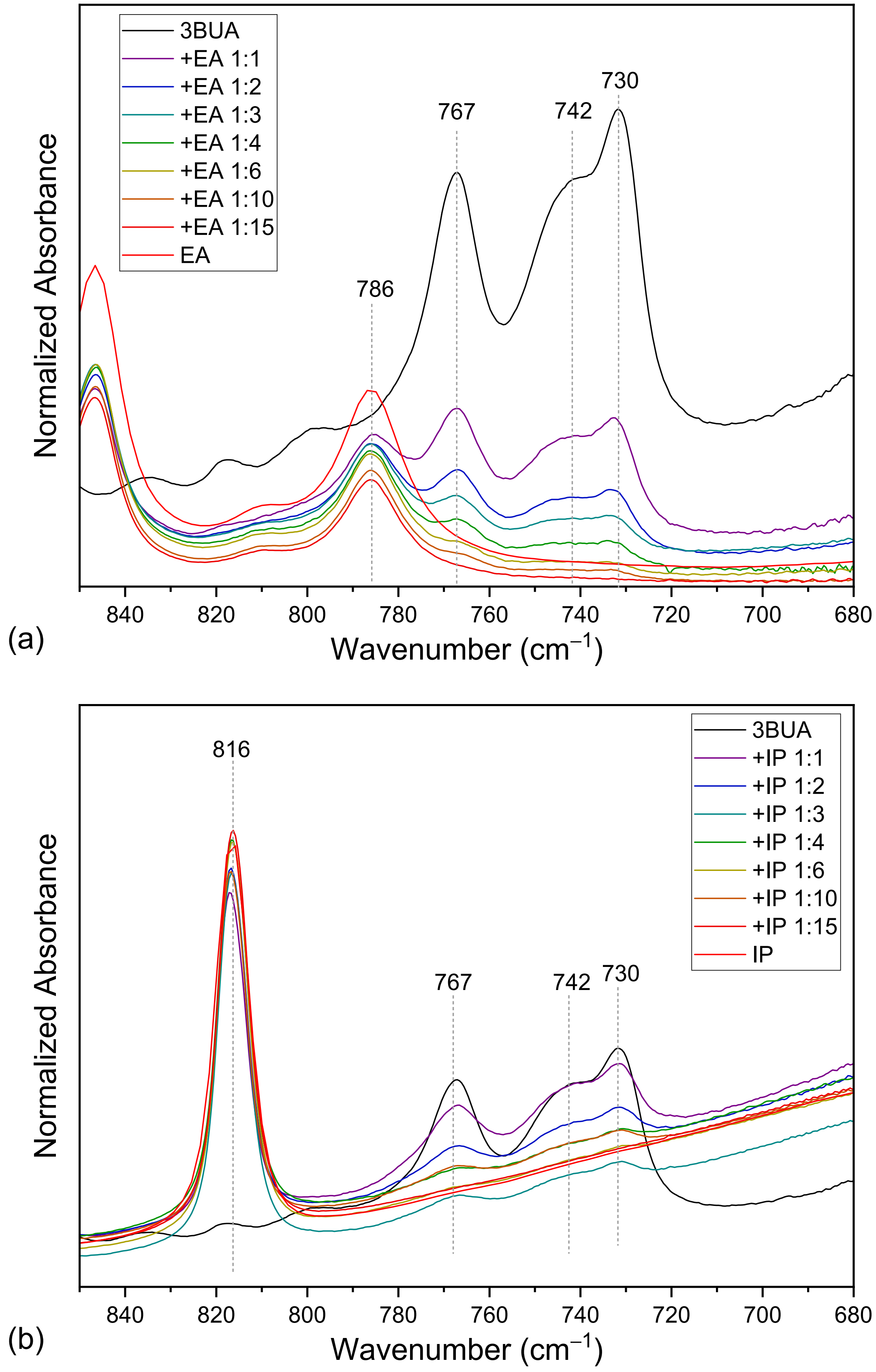

Figure S4. Urea skeletal vibration region of the IR spectra of 3BUA under increasing dilution with (a) EA and (b) IP.

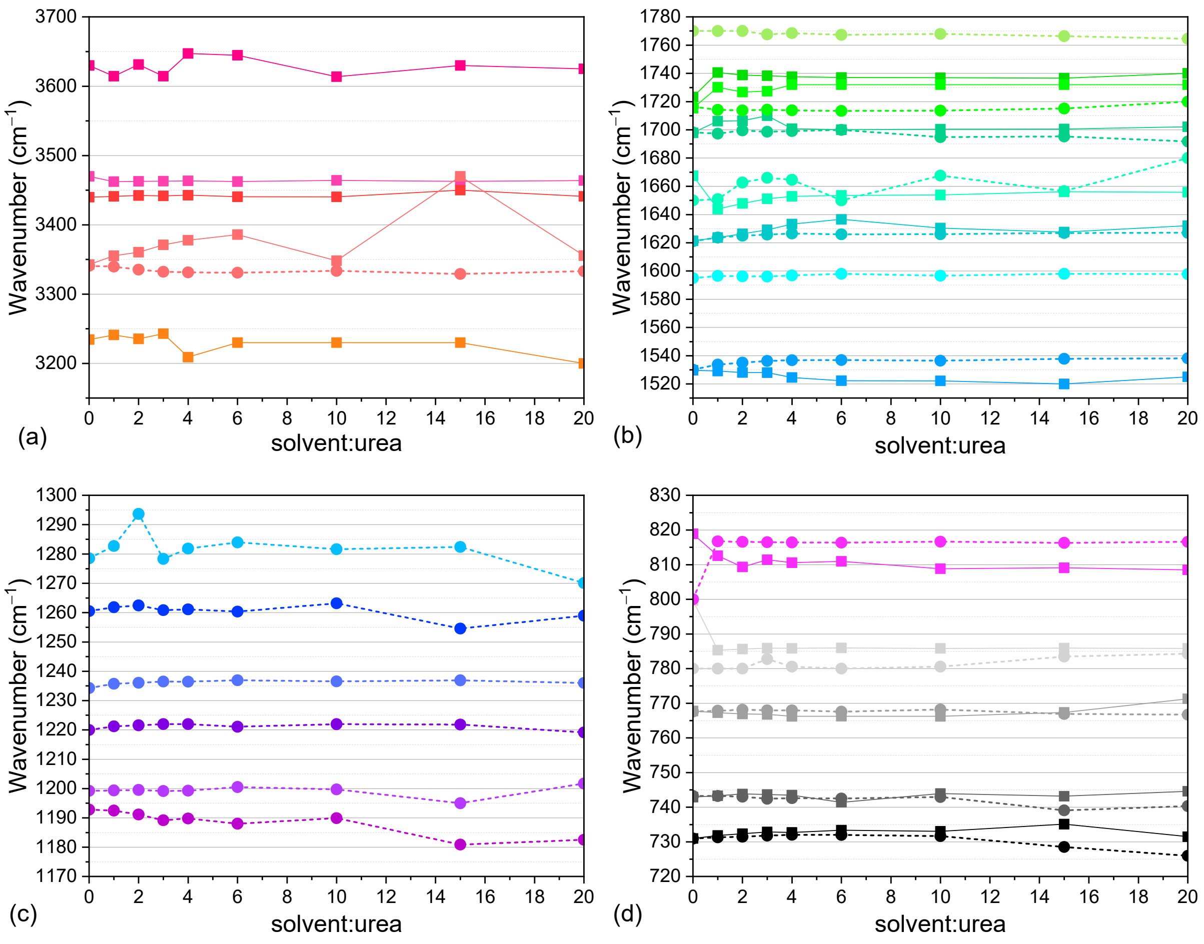

Figure S5. Evolution of the positions of selected IR bands with dilution with EA (solid lines) and IP (broken lines).

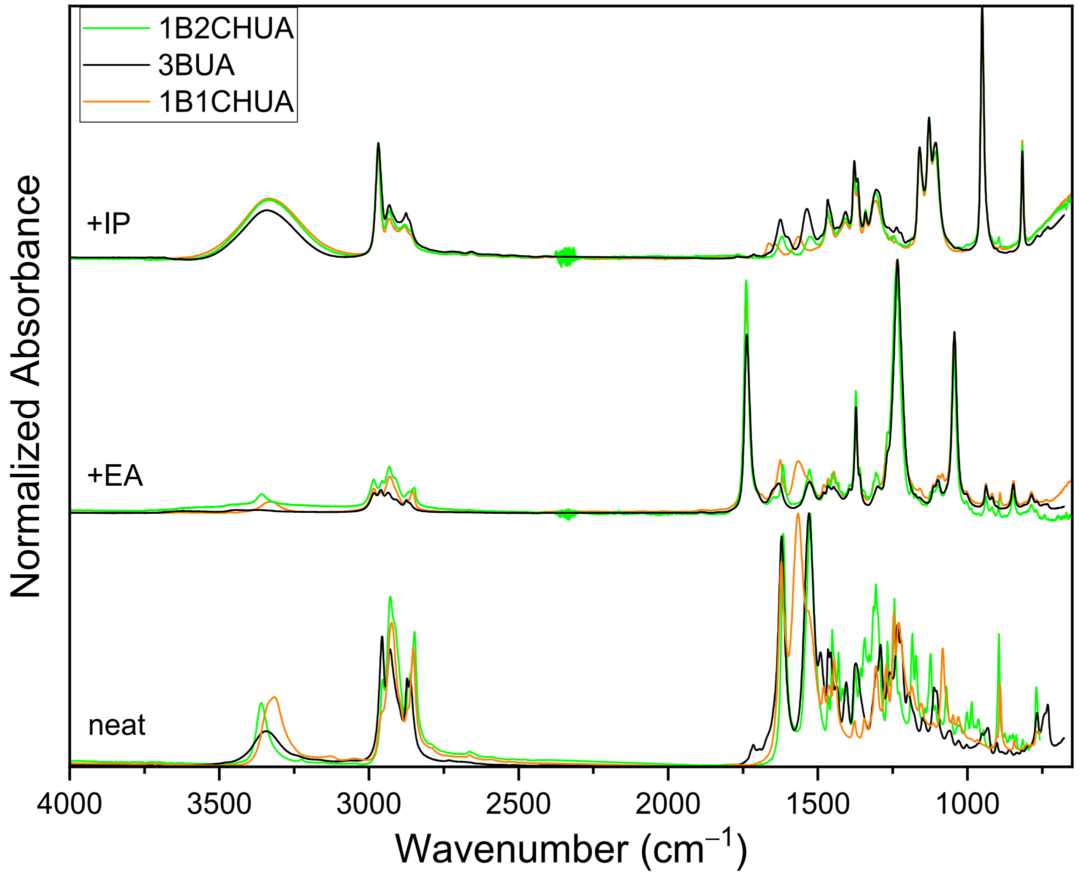

Figure S6. IR spectra of 3BUA, 1B2CHUA and 1B1CHUA neat and diluted with EA and IP.

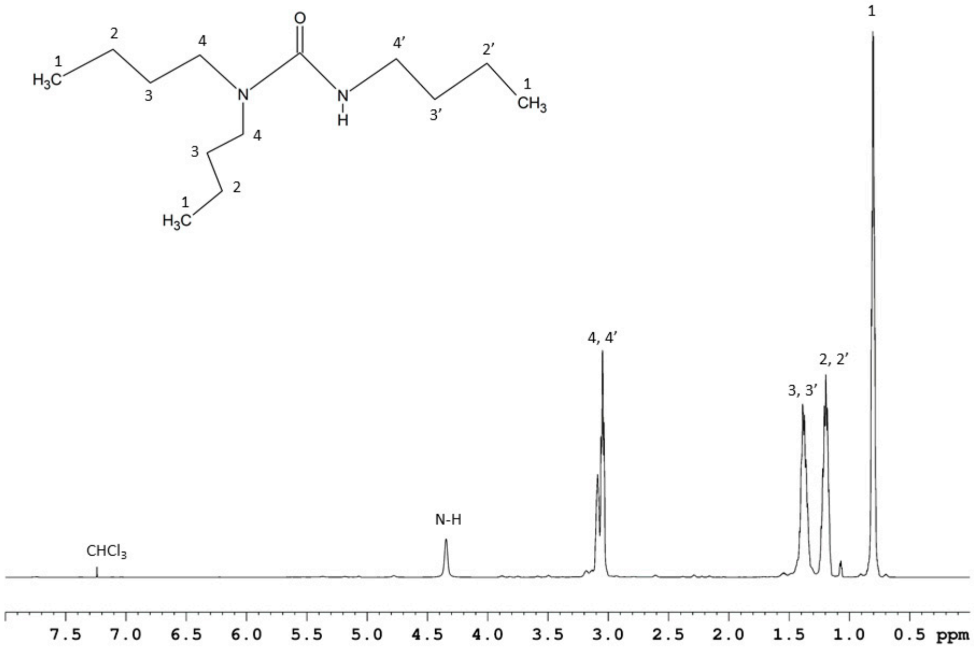

Figure S7. ^{1}H-NMR spectrum of 3BUA in $CHCl_3$.

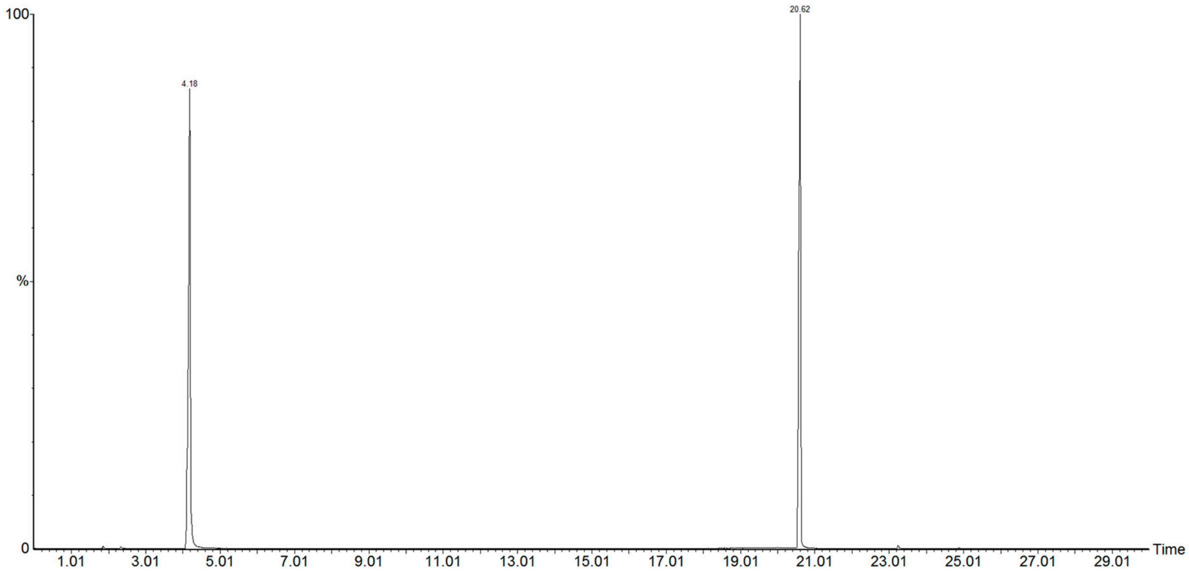

Figure S8. Chromatogram of the expected product (3BUA) in toluene solution.

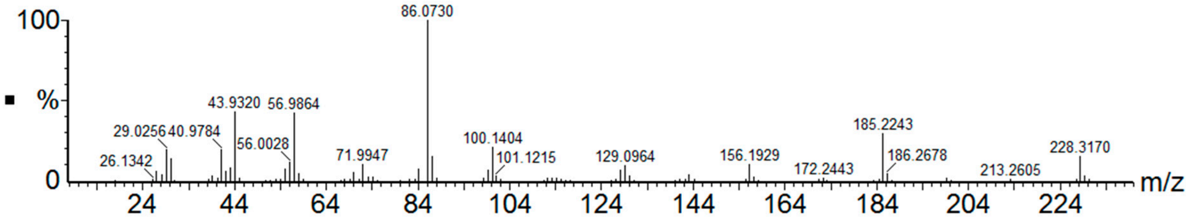

Figure S9. Mass spectrum of the peak identified as the expected product (3BUA).

The purity of the synthesized 3BUA was determined inTab toluene solution by gas chromatography coupled with mass spectrometry. Based on calculations from the areas of all detected peaks (excluding the solvent), the purity was determined to be 99.06 %.

S1 – specification of the fitting parameters in the structure x1=vlaue x of peak 1; x1b1= lower bound of x1; x1b2=upper bound of x1.
Full octave scripts are available as open data, DOI: XXX

Solutions in ethyl acetate

```
%region 1 - NH stretching
%peak centers
c1=3630;c1b1=3600;c1b2=3660;
c2=3460;c2b1=3450;c2b2=3470;
c3=3440;c3b1=3420;c3b2=3450;
c4=3345;c4b1=3320;c4b2=3470;
c5=3230;c5b1=3200;c5b2=3250;
%peak height
h1=0;h1b1=0;h1b2=inf;
h2=0;h2b1=0;h2b2=inf;
h3=0;h3b1=0;h3b2=inf;
h4=0.004;h4b1=0;h4b2=inf;
h5=0;h5b1=0;h5b2=inf;
%width of the gaussian component
wg1=7000;wg1b1=5000;wg1b2=10000;
wg2=500;wg2b1=200;wg2b2=5000;
wg3=500;wg3b1=200;wg3b2=5000;
wg4=1500;wg4b1=200;wg4b2=10000;
wg5=1500;wg5b1=500;wg5b2=20000;
%width of the lorentzian component
wl1=50;wl1b1=10;wl1b2=100;
wl2=15;wl2b1=5;wl2b2=50;
wl3=15;wl3b1=10;wl3b2=50;
wl4=25;wl4b1=10;wl4b2=150;
wl5=50;wl5b1=25;wl5b2=150;
%linear baseline
a=0.04;ab1=-inf;ab2=inf;
b=0;bb1=-1;bb2=1;

%region 2 - CH stretching
%peak centers
c1=2984;c1b1=2978;c1b2=2990;
c2=2960;c2b1=2950;c2b2=2970;
c3=2940;c3b1=2930;c3b2=2950;
c4=2905;c4b1=2890;c4b2=2920;
c5=2875;c5b1=2868;c5b2=2885;
c6=2860;c6b1=2850;c6b2=2868;
%peak height
h1=0.01;h1b1=0;h1b2=inf;
h2=0.01;h2b1=0;h2b2=inf;
h3=0.01;h3b1=0;h3b2=inf;
h4=0.01;h4b1=0;h4b2=inf;
```

```
h5=0.01;h5b1=0;h5b2=inf;
h6=0.01;h6b1=0;h6b2=inf;
%width of the gaussian component
wg1=50;wg1b1=10;wg1b2=700;
wg2=50;wg2b1=20;wg2b2=600;
wg3=50;wg3b1=20;wg3b2=600;
wg4=50;wg4b1=20;wg4b2=800;
wg5=50;wg5b1=20;wg5b2=600;
wg6=50;wg6b1=20;wg6b2=600;
%width of the lorentzian component
wl1=20;wl1b1=14;wl1b2=500;
wl2=20;wl2b1=10;wl2b2=800;
wl3=20;wl3b1=10;wl3b2=700;
wl4=20;wl4b1=10;wl4b2=600;
wl5=20;wl5b1=7;wl5b2=700;
wl6=20;wl6b1=10;wl6b2=600;
%linear baseline
a=0;ab1=-inf;ab2=inf;
b=0;bb1=-1;bb2=1;

%region 3 - carbonyl stretching
%peak centers
c1=1738;c1b1=1730;c1b2=1745;
c2=1727;c2b1=1720;c2b2=1732;
c3=1700;c3b1=1695;c3b2=1710;
c4=1655;c4b1=1635;c4b2=1680;
c5=1627;c5b1=1615;c5b2=1638;
%peak height
h1=0.1;h1b1=0;h1b2=inf;
h2=0;h2b1=0;h2b2=inf;
h3=0.01;h3b1=0;h3b2=inf;
h4=0.1;h4b1=0;h4b2=inf;
h5=0;h5b1=0;h5b2=inf;
%width of the gaussian component
wg1=100;wg1b1=25;wg1b2=200;
wg2=100;wg2b1=20;wg2b2=300;
wg3=150;wg3b1=20;wg3b2=500;
wg4=150;wg4b1=10;wg4b2=600;
wg5=150;wg5b1=10;wg5b2=500;
%width of the lorentzian component
wl1=10;wl1b1=5;wl1b2=15;
wl2=10;wl2b1=5;wl2b2=15;
wl3=15;wl3b1=5;wl3b2=20;
wl4=10;wl4b1=5;wl4b2=25;
wl5=10;wl5b1=5;wl5b2=25;
%linear baseline
a=0;ab1=-inf;ab2=inf;
```

b=0;bb1=-1;bb2=1;

%region 4 - amide II
%peak centers
c1=1530;c1b1=1520;c1b2=1550;
%peak height
h1=0.01;h1b1=0;h1b2=inf;
%width of the gaussian component
wg1=50;wg1b1=30;wg1b2=5000;
%width of the lorentzian component
wl1=7;wl1b1=3;wl1b2=50;
%linear baseline
a=0;ab1=-inf;ab2=inf;
b=0;bb1=-1;bb2=1;

%region 5 - urea skeletals
%peak centers
c1=810;c1b1=800;c1b2=830;
c2=785;c2b1=780;c2b2=805;
c3=767;c3b1=760;c3b2=775;
c4=743;c4b1=738;c4b2=750;
c5=732;c5b1=725;c5b2=738;
%peak height
h1=0.01;h1b1=0;h1b2=inf;
h2=0.01;h2b1=0;h2b2=inf;
h3=0.01;h3b1=0;h3b2=inf;
h4=0.01;h4b1=0;h4b2=inf;
h5=0.01;h5b1=0;h5b2=inf;
%width of the gaussian component
wg1=50;wg1b1=10;wg1b2=700;
wg2=50;wg2b1=10;wg2b2=600;
wg3=50;wg3b1=20;wg3b2=600;
wg4=50;wg4b1=20;wg4b2=600;
wg5=50;wg5b1=10;wg5b2=600;
%width of the lorentzian component
wl1=20;wl1b1=5;wl1b2=30;
wl2=20;wl2b1=5;wl2b2=80;
wl3=20;wl3b1=5;wl3b2=70;
wl4=10;wl4b1=5;wl4b2=60;
wl5=10;wl5b1=3;wl5b2=30;
%linear baseline
a=0;ab1=-inf;ab2=inf;
b=0;bb1=-1;bb2=1;

Solutions in isopropanol
%region 1 - NH stretching
c1=3340;c1b1=3300;c1b2=3400;

```
%peak height
h1=0.01;h1b1=0;h1b2=inf;
%width of the gaussian component
wg1=7000;wg1b1=5000;wg1b2=25000;
%width of the lorentzian component
wl1=50;wl1b1=10;wl1b2=150;
%linear baseline
a=0.04;ab1=-inf;ab2=inf;
b=0;bb1=-1;bb2=1;

%region 2 - CH stretching
%peak centers
c1=2970;c1b1=2965;c1b2=2980;
c2=2956;c2b1=2950;c2b2=2960;
c3=2930;c3b1=2925;c3b2=2935;
c4=2895;c4b1=2890;c4b2=2920;
c5=2880;c5b1=2865;c5b2=2885;
c6=2862;c6b1=2855;c6b2=2868;
%peak height
h1=0;h1b1=0;h1b2=inf;
h2=0.01;h2b1=0;h2b2=inf;
h3=0.01;h3b1=0;h3b2=inf;
h4=0.01;h4b1=0;h4b2=inf;
h5=0.01;h5b1=0;h5b2=inf;
h6=0.01;h6b1=0;h6b2=inf;
%width of the gaussian component
wg1=50;wg1b1=10;wg1b2=700;
wg2=50;wg2b1=10;wg2b2=600;
wg3=50;wg3b1=20;wg3b2=600;
wg4=50;wg4b1=20;wg4b2=1000;
wg5=50;wg5b1=20;wg5b2=600;
wg6=50;wg6b1=20;wg6b2=600;
%width of the lorentzian component
wl1=20;wl1b1=10;wl1b2=500;
wl2=20;wl2b1=7;wl2b2=800;
wl3=20;wl3b1=10;wl3b2=700;
wl4=20;wl4b1=10;wl4b2=600;
wl5=20;wl5b1=7;wl5b2=700;
wl6=50;wl6b1=10;wl6b2=600;
%linear baseline
a=0;ab1=-inf;ab2=inf;
b=0;bb1=-1;bb2=1;

%region 3 - carbonyl stretching
%peak centers
c1=1767;c1b1=1760;c1b2=1770;
c2=1715;c2b1=1710;c2b2=1720;
```

```
c3=1695;c3b1=1690;c3b2=1700;
c4=1655;c4b1=1650;c4b2=1680;
c5=1627;c5b1=1615;c5b2=1638;
c6=1600;c6b1=1590;c6b2=1610;
%peak height
h1=0;h1b1=0;h1b2=inf;
h2=0;h2b1=0;h2b2=inf;
h3=0;h3b1=0;h3b2=inf;
h4=0.005;h4b1=0;h4b2=inf;
h5=0.02;h5b1=0;h5b2=inf;
h6=0.005;h6b1=0;h6b2=inf;
%width of the gaussian component
wg1=100;wg1b1=25;wg1b2=200;
wg2=100;wg2b1=20;wg2b2=300;
wg3=150;wg3b1=20;wg3b2=500;
wg4=800;wg4b1=20;wg4b2=1000;
wg5=150;wg5b1=10;wg5b2=600;
wg6=150;wg6b1=10;wg6b2=1000;
%width of the lorentzian component
wl1=10;wl1b1=5;wl1b2=15;
wl2=10;wl2b1=5;wl2b2=15;
wl3=15;wl3b1=5;wl3b2=20;
wl4=10;wl4b1=5;wl4b2=25;
wl5=10;wl5b1=5;wl5b2=25;
wl6=15;wl6b1=5;wl6b2=35;
%linear baseline
a=-0.01;ab1=-inf;ab2=inf;
b=0;bb1=-1;bb2=1;

%region 4 - amide II
%peak centers
c1=1530;c1b1=1520;c1b2=1550;
%peak height
h1=0.01;h1b1=0;h1b2=inf;
%width of the gaussian component
wg1=50;wg1b1=30;wg1b2=5000;
%width of the lorentzian component
wl1=7;wl1b1=3;wl1b2=50;
%linear baseline
a=0;ab1=-inf;ab2=inf;
b=0;bb1=-1;bb2=1;

%region 5 - alcohol C-O stretching
%peak centers
c1=1290;c1b1=1270;c1b2=1350;%band in neighboring region creating baseline
c2=1260;c2b1=1250;c2b2=1270;
c3=1235;c3b1=1230;c3b2=1240;
```

```
c4=1222;c4b1=1215;c4b2=1230;
c5=1200;c5b1=1195;c5b2=1205;
c6=1190;c6b1=1180;c6b2=1195;
%peak height
h1=0.01;h1b1=0.0005;h1b2=inf;
h2=0;h2b1=0;h2b2=inf;
h3=0;h3b1=0;h3b2=inf;
h4=0;h4b1=0;h4b2=inf;
h5=0;h5b1=0;h5b2=inf;
h6=0.01;h6b1=0;h6b2=inf;
%width of the gaussian component
wg1=1000;wg1b1=300;wg1b2=2000;
wg2=50;wg2b1=10;wg2b2=1000;
wg3=50;wg3b1=10;wg3b2=600;
wg4=50;wg4b1=20;wg4b2=600;
wg5=50;wg5b1=10;wg5b2=600;
wg6=150;wg6b1=10;wg6b2=500;
%width of the lorentzian component
wl1=20;wl1b1=5;wl1b2=30;
wl2=20;wl2b1=5;wl2b2=80;
wl3=20;wl3b1=5;wl3b2=70;
wl4=10;wl4b1=5;wl4b2=60;
wl5=10;wl5b1=3;wl5b2=30;
wl6=10;wl6b1=3;wl6b2=25;
%linear baseline
a=0.03;ab1=-inf;ab2=inf;
b=0;bb1=-1;bb2=1;

%region 6 - urea skeletals
%peak centers
c1=810;c1b1=800;c1b2=830;
c2=785;c2b1=780;c2b2=805;
c3=767;c3b1=760;c3b2=775;
c4=743;c4b1=738;c4b2=750;
c5=732;c5b1=725;c5b2=738;

%peak height
h1=0.01;h1b1=0;h1b2=inf;
h2=0.01;h2b1=0;h2b2=inf;
h3=0.01;h3b1=0;h3b2=inf;
h4=0.01;h4b1=0;h4b2=inf;
h5=0.01;h5b1=0;h5b2=inf;

%width of the gaussian component
wg1=50;wg1b1=10;wg1b2=700;
wg2=50;wg2b1=10;wg2b2=600;
wg3=50;wg3b1=20;wg3b2=600;
```

```
wg4=50;wg4b1=20;wg4b2=600;
wg5=50;wg5b1=10;wg5b2=600;

%width of the lorentzian component
wl1=20;wl1b1=5;wl1b2=30;
wl2=20;wl2b1=5;wl2b2=80;
wl3=20;wl3b1=5;wl3b2=70;
wl4=10;wl4b1=5;wl4b2=60;
wl5=10;wl5b1=3;wl5b2=30;

%linear baseline
a=0;ab1=-inf;ab2=inf;
b=0;bb1=-1;bb2=1;
```
